# A rapid method to authenticate vegetable oils through surface-enhanced Raman scattering

**DOI:** 10.1038/srep23405

**Published:** 2016-03-18

**Authors:** Ming Yang Lv, Xin Zhang, Hai Rui Ren, Luo Liu, Yong Mei Zhao, Zheng Wang, Zheng Long Wu, Li Min Liu, Hai Jun Xu

**Affiliations:** 1 Beijing Bioprocess Key Laboratory, Beijing University of Chemical Technology, Beijing, 100029, PR China; 2 College of Science, Beijing University of Chemical Technology, Beijing, 100029, PR China; 3 Engineering Research Center for Semiconductor Integrated Technology, Institute of Semiconductors, Chinese Academy of Sciences, Beijing, 100083, PR China; 4 Analytical and Testing Center, Beijing Normal University, Beijing, 100875, PR China

## Abstract

Vegetable oils are essential in our daily diet. Among various vegetable oils, the major difference lies in the composition of fatty acids, including unsaturated fatty acids (USFA) and saturated fatty acids (SFA). USFA include oleic acid (OA), linoleic acid (LA), and α-linolenic acid (ALA), while SFA are mainly palmitic acid (PA). In this study, the most typical and abundant USFA present with PA in vegetable oils were quantified. More importantly, certain proportional relationships between the integrated intensities of peaks centered at 1656 cm^−1^ (S_1656_) in the surface-enhanced Raman scattering spectra of different USFA were confirmed. Therefore, the LA or ALA content could be converted into an equivalent virtual OA content enabling the characterization of the USFA content in vegetable oils using the equivalent total OA content. In combination with the S_1656_ of pure OA and using peanut, sesame, and soybean oils as examples, the ranges of S_1656_ corresponding to the National Standards of China were established to allow the rapid authentication of vegetable oils. Gas chromatograph-mass spectrometer analyses verified the accuracy of the method, with relative errors of less than 5%. Moreover, this method can be extended to other detection fields, such as diseases.

Raman spectroscopy is a powerful and highly sensitive analytical technique yielding individual molecular spectra (fingerprints). It is now increasingly being applied in various fields from fundamental research to engineering in physics, biology, environmental science, and chemistry^1^,^2^,^3^,^4^,^5^,^6^. However, it has only acted as a supplement to techniques using the infrared spectrum because of its low efficiency, as only one of every 10^8^ incident photons is scattered to produce a Raman signal. Early work in the 1970s on Raman scattering using pyridine molecules on the rough surface of a silver electrode found that the Raman signal was enhanced one million times, an effect known as surface-enhanced Raman scattering (SERS)^7^. Since then, because of its combined advantages of rapid response, non-destructive analysis, high sensitivity, and even single molecule detection^8^,^9^,^10^, SERS has been widely applied.

Being concerned with their health, consumers have become increasingly interested in food composition, particularly fatty acid (FA) contents. Vegetable oils, rich in essential FAs, are important in our daily diet^11^. The main FAs in vegetable oils are oleic acid (OA), linoleic acid (LA), α-linolenic acid (ALA), and palmitic acid (PA)^12^. Of these, PA is a saturated fatty acid (SFA) containing no C=C bonds, while OA, LA, and ALA are all unsaturated fatty acids (USFA) containing 1, 2 and 3 C=C bonds, respectively^13^,^14^. USFA are important because of their role in several physiological processes affecting normal health and chronic diseases: the regulation of plasma lipid levels, cardiovascular and immune functions, insulin action and neuronal development and visual function^15^. Nevertheless, unscrupulous manufacturers may sell vegetable oils, adulterated with large amounts of cheap oils or completely counterfeit greatly altering the USFA content. Consequently, a low-cost, rapid, and universal method for detecting the USFA content is required to ensure the quality of vegetable oils. Several techniques have been developed to authenticate vegetable oils, such as the iodine value (IV) method^13^, gas chromatography (GC)^16^,^17^, and gas chromatography-mass spectrometry (GC-MS)^18^. However, the standard IV determination using classical titration is time consuming and inaccurate, and the GC and GC-MS techniques not only require complicated and expensive laboratory facilities but also involve complicated procedures. SERS spectroscopy, which is sensitive, low-cost, and rapid, may overcome these disadvantages. In combination with principal component analysis (PCA), partial least-squares, least squares support vector machines (LS-SVM), or neural network techniques^19^,^20^,^21^,^22^, Raman spectroscopy has been successfully used to authenticate oils: Zou *et al.* detected olive oil adulteration using the Raman spectrum combined with two-dimensional figure and PCA^23^; and Dong *et al.* predicted the fatty acid composition of vegetable oils based on Raman spectroscopy and the LS-SVM technique^20^. Although these detection methods can establish standards to authenticate oils with a high level of accuracy, they also require knowledge of chemometrics and sophisticated data processing, which limits their widespread application.

The present study aims to establish a novel and universal analysis system based on SERS to authenticate whether the USFA content of vegetable oils conforms to certain quality control specifications, such as those of the National Standard of China (GB). The conversion relationship based on the ratio between the integrated SERS intensity of the peak centered at 1656 cm^−1^ (S_1656_) will be found for OA and for LA and ALA. Consequently, the LA or ALA content can be converted into an equivalent virtual OA content, so the USFA content of the vegetable oils can thus be characterized by an equivalent total content (ETC) of OA that combines the actual and virtual contents. Using the S_1656_ value of pure OA with those of peanut, sesame, and soybean oils as examples, the allowable ranges of S_1656_ value corresponding to GB will be established as box plots. This will identify rapidly whether the USFA content of these vegetable oils conforms to the GB standards. Furthermore, the universality of this method can be exploited not only to authenticate vegetable oils, but also to authenticate the degree of unsaturation in cells and plasma. In our study, the silver/silicon (Ag/Si) substrate will be used for the SERS test.

## Results and Discussion

### SERS spectra of FAS

**Figure 1:**
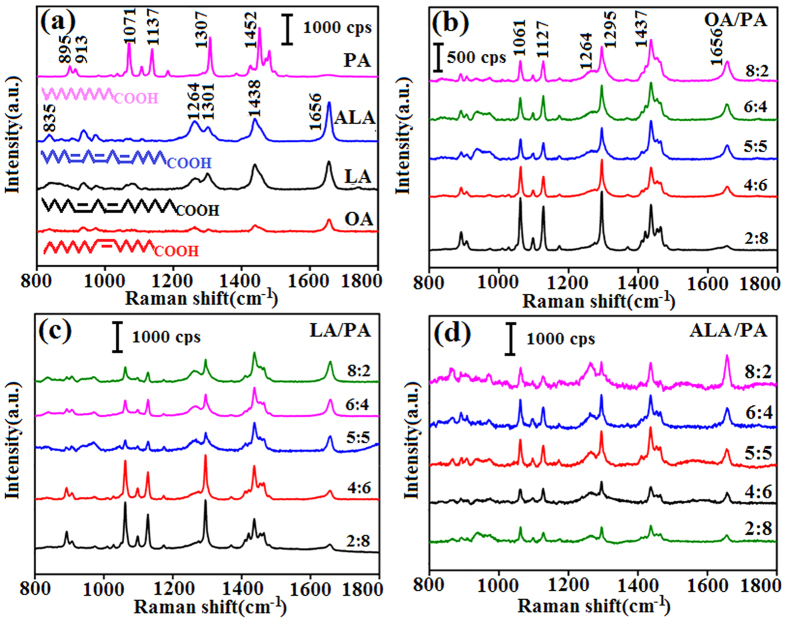
(**a**) SERS spectra and molecular structures of PA, OA, LA, and ALA. SERS spectra of (**b**) OA/PA, (**c**) LA/PA, and (**d**) ALA/PA with different molar ratios.

**Figure 2:**
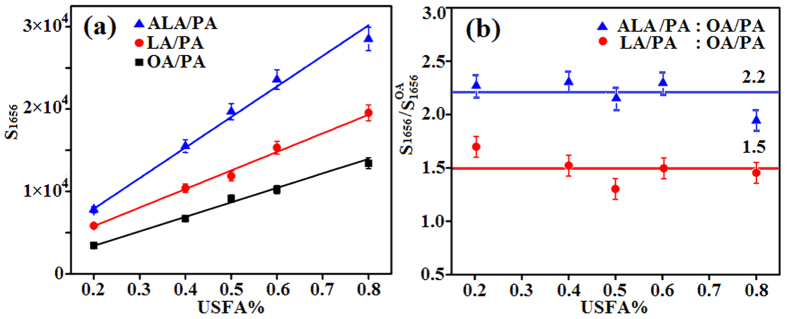
(**a**) Response curve between S_1656_ value and the USFA content for the OA/PA, LA/PA, and ALA/PA samples. (**b**) Ratios of S_1656_ values for LA/PA and ALA/PA to OA/PA at the same USFA content; horizontal lines - mean values of the ratios; and 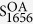 represents the S_1656_ value of OA/PA. Error bars are marked.

Four groups of mixed samples, OA/PA, LA/PA, ALA/PA, and OA/ALA, were prepared for our experiment, with the mole ratio of the two fatty acids in each group being 2:8, 4:6, 5:5, 6:4 and 8:2, respectively. Pure PA is solid and does not dissolve completely in liquid USFA at room temperature. To overcome this problem, the mixtures were placed in airtight centrifuge tubes warmed in hot water at 70 °C. After the PA has melted and dissolved thoroughly in the USFA, the mixed samples were taken out using a pipetting gun and dropped onto the SERS Ag/Si substrate. Using a randomly selected 5:5 OA/PA sample, the SERS and Raman spectra were obtained using an Ag/Si substrate and a Si wafer, respectively (seeing Supplementary Fig. S1). The superiority of SERS was obvious, so all the following tests were performed on Ag/Si substrates. Figure 1a shows the SERS spectra of four types of pure FA. The molecular formulas of OA, LA, ALA, and PA are all extremely simple containing only -CH_3_, -CH_2_, and -COOH as functional groups and C=C, -C-C-, -C-H, O=C-, -OH, and C-O as chemical bonds^24^. PA, an SFA, contains 16 carbon atoms and no C=C bond. Characteristically, PA has Raman bands between 1050 and 1150 cm^−1^, a band at 1307 cm^−1^, and a group of bands in the region between 1400 and 1500 cm^−1^, owing to υ(C-C) stretching vibrations, ζ(CH_2_) twist vibrations, and ζ(CH_3_) or ζ(CH_2_) deformations, respectively^25^,^26^. OA, LA, and ALA are USFA with 1, 2 and 3 C=C bonds, respectively. Their molecular formulas include the same number of carbon atoms and the same type of functional groups but different numbers of C=C bonds, therefore, their SERS spectra are similar. The characteristic peaks for OA, LA and ALA are the band at 1656 cm^−1^ owing to υ(C=C) stretching vibrations, the band at 835 cm^−1^ assigned to υ(C-C) stretching vibrations, the band at 1264 cm^−1^ assigned to the in-plane CH bending deformations from the cis-double bonds coupling to the CH_2_ rocking modes, the bands at 1301 and 1438 cm^−1^ ascribed to ζ(CH_2_)_n_ deformations and the CH_2_ scissoring^24^,^27^,^28^. The band centered at 939 cm^−1^ is assigned to the bare silicon in the Ag/Si substrate. Furthermore, USFA are fluids and their SERS spectra show broader bands compared with SFA. Vegetable oils consist mainly of OA, LA, ALA, and PA, so PA was used as the solvent with OA, LA, and ALA as the solutes to simulate the environment of vegetable oils. The SERS spectra of OA/PA, LA/PA, and ALA/PA are shown in Fig. 1b–d, respectively. The intensity of the bands centered at 1656 cm^−1^ became obviously stronger as the USFA content increased or in other words, a higher concentration of C=C bonds led to a stronger SERS signal. Additionally, the Raman shift of some characteristic peaks of FAs changed because of mixing with another type of FA. For example, the bands for PA centered at 1071 and 1137 cm^−1^ shifted to 1061 and 1127 cm^−1^, respectively; and the band for PA centered at 1307 cm^−1^ and that for OA at 1301 cm^−1^ merged and shifted to 1295 cm^−1^. Figure 2a shows the relationship between S_1656_ and the USFA content for the three groups of samples in Fig. 1b–d. The S_1656_ values of pure USFA have also been plotted as a special case (seeing Supplementary Fig. S2).Using this curve, the content of USFA can be quantified if only one type of USFA is present. However, most oils contain at least two types of USFA, therefore, further analysis is needed.

### Conversion relationship analysis

To discover the relationship between the S_1656_ values corresponding to OA and to LA/ALA, the S_1656_ values of OA/PA at five different ratios (2:8, 4:6, 5:5, 6:4, and 8:2) were used as benchmarks, and those of LA/PA and ALA/PA were divided by these benchmarks at the same USFA content (Fig. 2b). The S_1656_ values of LA/PA and ALA/PA were about 1.5 and 2.2 times that of OA/PA, respectively, and very similar for different molar ratios. Thus, the S_1656_ values of LA and ALA are about 1.5 and 2.2 times respectively of OA, considering that PA contributes nothing to S_1656_ owing to its molecular structure. This result is not consistent with the number of C=C bonds in the OA, LA, and ALA molecules because the vibration of the C=C bond is not completely isolated and will be affected by other functional groups in the molecule itself and even by other molecules. Therefore, using the quantitative factors of 1.5 and 2.2, the contents of LA and ALA in vegetable oils can be converted to that of virtual OA, leading to simplifying the analysis process and thus detecting the USFA content of vegetable oils.

**Figure 3:**
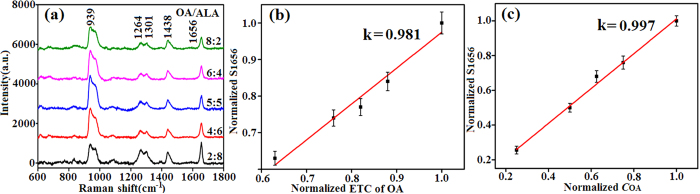
(**a**) SERS spectra of OA/ALA with different molar radios. (**b**) Relationship between the normalized S_1656_ value and the normalized ETC of OA corresponding to (**a**). (**c**) Relationship between the normalized S_1656_ value and the normalized OA content corresponding to Fig. 1(b). Error bars are marked.

The SERS spectra of OA/ALA were also measured to validate the accuracy of this conversion relationship. Figure 3a shows the SERS spectra of OA/ALA at different molar ratios. As expected, the SERS signal at 1656 cm^−1^ weakened as the OA content increased. The S_1656_ values and corresponding ETC of OA are listed in Supplemental Table S1 with their normalized values. The ETC of OA refers to the sum of the actual OA content in the sample and the virtual equivalent OA content obtained as the product of the ALA content and the conversion relationship of 2.2. Taking the samples with molar ratios of 2:8 and 4:6 as examples, their ETC of OA values are 20% + 80% × 2.2 = 1.96 and 40% + 60% × 2.2 = 1.72, respectively. Figure 3b shows the linear relationship between the normalized S_1656_ values and the normalized ETC of OA, which has a slope of 0.981. For comparison, the relationship corresponding to Fig. 1b, which contains OA as the only USFA, was normalized to Fig. 3c. Its slope was 0.997, close to 0.981. These results indicate that the ETC of OA plays the same role as OA content in contributing to the S_1656_ value, therefore validating the accuracy of the conversion relationship. In addition, as the proportional coefficient is approximately 1, the S_1656_ values can be calculated by the product of the ETC of OA and the S_1656_ value of pure OA.

### Authentication of vegetable oils

For varieties of vegetable oils sold in the market, the USFA contents are an important indication of their authenticity and quality. A quantitative appraisal method based on the above conversion relationships has been proposed in the present study to validate whether the USFA contents of vegetable oils conform to GB standards. Initially, the threshold range of the ETC of OA should be calculated for each type of oil regulated in the standard. The allowable range of S_1656_ corresponding to the standard can then be determined by combining this with the S_1656_ value of pure OA. To authenticate any oil on the market, its S_1656_ value should be tested and compared with the calculated allowable range. To assess this appraisal method, it will be applied to three types of common vegetable oils sold in China, peanut, sesame, and soybean oils. Table 1 lists the corresponding National Standards of China for these oils (Peanut oil, GB 1534–2003; Sesame oil: GB 8233–2008; Soybean oil: GB 1535–2003), where the notation in the first column represents the type of FA. Specifically, C_16:0_ represents PA that contains 16 carbon atoms and no C=C bonds; C_18:1_ represents OA containing 18 carbon atoms and one C=C bond; C_18:2_, and C_18:3_ represents LA and ALA, respectively, in a similar way.

**Figure 4:**
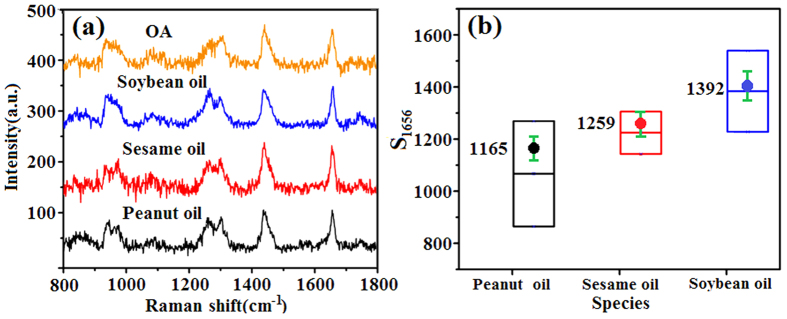
(**a**) SERS spectra of pure OA and three types of vegetable oil (soybean, sesame, and peanut). (**b**) Box plots of S_1656_ corresponding to the ETC of OA, with the measured S_1656_ values of three types of vegetable oils (Luhua brand). Error bars are marked.

To describe the calculations clearly, peanut oil in Table 1 is taken as an example. For the upper limit of the ETC of OA, the number of C=C bonds should reach the maximum, which requires the USFA to take the minimum value in the allowable range. Therefore, C_16:0_, C_18:0_, C_20:0_, C_22:0_, and C_24:0_ should be 8.0, 1.0, 1.0, 1.5, and 0.5 g, respectively, a total of 12.0 g. At the same time, the USFA of C_18:3_ (ALA) and C_18:2_ (LA) should take the maximum value of 0.3 g and 43.0 g, respectively, and thus C_18:1_ (OA) is 44.7 g (100 − 12 − 0.3 − 43 = 44.7). After converting grams to moles and combining with the conversion relationships of 1.5 and 2.2, the upper limit of the ETC of OA corresponding to GB can be calculated. In contrast, when the contents of USFA are maximal, i.e., C_16:0_, C_18:0_, C_20:0_, C_22:0_, and C_24:0_ are 14.0, 4.5, 2.0, 4.5, and 2.5 g, respectively (a total of 27.5 g), and when C_18:1_ takes the maximum value of 69.0 g then C_18:2_ is 3.5 g (100 − 27.5 − 69 = 3.5), the lower limit of the ETC of OA can be calculated. The results for the other vegetable oils can be obtained similarly (Table 2). Column B shows the products of the values in column A and the measured S_1656_ value of pure OA (1166, as shown in Fig. 4a), which represents the allowable ranges of S_1656_ corresponding to GB standards. Column C lists the S_1656_ values of the three types of vegetable oils according to Fig. 4a. It should be pointed out that the SERS spectra in Fig. 4a were obtained using a laser excitation power of 0.35 mW to avoid degenerating the samples by high temperatures. The box plots shown in Fig. 4b were plotted based on the results from Table 2. Their upper and lower edges correspond to the values in column A and B, respectively and the points to those in column C. As the three points are all within the boxes, this confirms that the USFA contents of all three types of vegetable oil conformed to GB standards. If the S_1656_ value of a vegetable oil had fallen outside the box, its USFA content would not have conformed. Thus, vegetable oils can be authenticated rapidly.

### Validation

GC-MS analysis has also been used to confirm the FA contents of the three types of vegetable oil (Table 1). The results revealed that all FA contents conformed to the National Standards of China. The corresponding S_1656_ values were further calculated (Table 2). Obviously, these values are very close to those in column C, with relative errors of less than 5%, indicating the accuracy of an appraisal method based on SERS. The relative error for peanut oil was a little higher than those for the other two oils, which may be because the C_20:1_ content was ignored during the calculation. Therefore, this novel method has been validated by our experiments as effective for authenticating vegetable oils. This method can not only be used to authenticate the three types of oil tested, but could also be expanded to detect USFA in other types of oil by establishing the relationship between the corresponding National Standard and the measured S_1656_ value of the oils of interest. An accumulation time as short as 15 s ensures the quickness of detection. Thus, it could be of great significance for monitoring the edible oil market and ensuring food safety and quality.

## Conclusion

To summarize, a novel, quick, and universal method to authenticate whether the USFA content in vegetable oils conforms to the National Standards of China has been established. Analysis using SERS spectra has shown the S_1656_ values of LA and ALA to be 1.5 and 2.2 times that of OA under the same experimental conditions. Based on this conversion relationship, the USFA content of peanut, sesame, and soybean oils specified in the National Standards of China were converted to the ETC of OA. Then, by using the S_1656_ value of pure OA, box plots of S_1656_ corresponding to the National Standards of China of the three types of vegetable oils have been depicted for oil authentication. The GC-MS analyses further verified the accuracy of the method, with relative errors of less than 5%. It is worth noting that this method can be applied to authenticate any type of vegetable oil containing USFA. In addition, the conversion relationship obtained in this study for FAs could also be applied to other fields. For example, analyzing the USFA content in cell membrane and plasma may help detect several diseases in advance.

## Methods

### Materials and instruments

HF, H_2_O_2_, AgNO_3_, OA, LA, ALA, and PA were purchased from J&K Scientific Ltd. Peanut, sesame, and soybean oils (Luhua brand) were purchased from a well-known supermarket in China. Deionized water (18 MΩ) (Beijing Chemical Works, Beijing, China) was used for all experiments. All chemicals, unless mentioned otherwise, were of analytical grade and used as received.

All the Raman signals were obtained at room temperature using a LabRAM ARAMIS Raman system with a 785-nm laser for excitation. The spectral resolution was 1 cm^−1^ and the diameter of the light spot ~1 μm. The laser excitation power was 3.5 mW in the case of no special instructions. All spectra were recorded using an accumulation time of 15 s. The data were averaged over 10 randomly selected positions and the error bars calculated. All the samples including PA were detected after melting in hot water.

### Substrate preparation

The Ag/Si substrate was prepared using the method described by Zhang *et al.*^29^. The synthesis was carried out at room temperature. N-type (100) Si wafers (1–10 Ω cm) were used as the substrate material and cut into pieces (1 × 3 cm). The cut Si wafer was cleaned sequentially in deionized water, acetone, and alcohol for 5 min each. It was then immersed in a solution of 4.6 M HF and 0.005 M AgNO_3_ for 2 min and washed with deionized water several times. Then, the template was placed in a mixture of 4.6 M HF and 0.5 M H_2_O_2_ for 60 min. Finally, the template was immersed in a solution of 0.01 M AgNO_3_ until the surface became milky white. The substrates were characterized by field emission scanning electron microscopy and transmission electron microscopy measurements (seeing Supplementary Fig. S3).

## Additional Information

**How to cite this article**: Lv, M. Y. *et al.* A rapid method to authenticate vegetable oils through surface-enhanced Raman scattering. *Sci. Rep.* **6**, 23405; doi: 10.1038/srep23405 (2016).

## Supplementary Material

Supplementary Information

## Figures and Tables

**Table 1:** Fatty acid composition of three types of vegetable oil as defined by the National Standards of China (GB) and as quantified by GC-MS analysis of corresponding Luhua brand vegetable oils (/100 g)

Fatty acids	GB			GC-MS		
	Peanut	Sesame	Soybean	Peanut	Sesame	Soybean
C16:0	8.0−14.0	7.9−12.0	8.0−13.5	11.6	10.0	12.1
C18:0	1.0−4.5	4.5−6.7	2.0−5.4	3.0	5.8	4.5
C18:1	35.0−69.0	34.4−45.5	17.0−30.0	37.2	37.8	24.0
C18:2	12.0−43.0	36.9−47.9	48.0−59.0	38.8	44.6	50.4
C18:3	ND−0.3	0.2−1.0	4.5−11.0	0.1	0.4	7.5
C20:0	1.0−2.0	0.3−0.7	0.1−0.6	1.6	0.7	0.4
C20:1	0.7−1.7	ND−0.3	ND−0.5	1.4	0.2	0.2
C22:0	1.5−4.5	ND−1.1	ND−0.7	4.0	0.2	0.4
C24:0	0.5−2.5	ND−0.3	ND−0.5	1.9	0.1	0.1
Sum				99.6	99.8	99.6

ND-not detected, content < 0.05%.

**Table 2:** Allowable ranges of S_1656_ according to National Standards of China, together with the measured and calculated S_1656_ of three types of Luhua brand vegetable oils.

Sample (oil)	A		B		C	GC-MSCalculated	ξ (%)
	Max	Min	Max	Min	Measured		
Peanut	1.09	0.742	1270	865	1165 ± 46	1112	4.77
Sesame	1.12	0.98	1306	1143	1259 ± 47	1228	2.52
Soybean	1.32	1.053	1539	1228	1392 ± 57	1357	2.58

Relative errors are calculated.
