# Supplementary material for: A rapid method to authenticate vegetable oils through surface-enhanced Raman scattering
Source: Sci Rep. 2016 Mar 18;6:23405. doi: 10.1038/srep23405 (PMC4796845; doi:10.1038/srep23405)
Supplement: Supplementary Information [file srep23405-s1.pdf]

Supplementary Material

A rapid method to authenticate vegetable oils through surface-enhanced Raman scattering

Ming Yang Lv[a,b], Xin Zhang[a,b,]*, Hai Rui Ren[a,b], Luo Liu[a], Yong Mei Zhao[c], Zheng Wang[a], Zheng Long Wu[d], Li Min Liu[b], Hai Jun Xu[a,b,]*

[a]Beijing Bioprocess Key Laboratory, Beijing University of Chemical Technology, Beijing, 100029, PR China

[b]College of Science, Beijing University of Chemical Technology, Beijing, 100029, PR China

[c]Engineering Research Center for Semiconductor Integrated Technology, Institute of Semiconductors, Chinese Academy of Sciences, Beijing, 100083, PR China

[d]Analytical and Testing Center, Beijing Normal University, Beijing, 100875, PR China

* Corresponding authors:

Tel.: +86-10-64414824; Fax: +86-10-64435706

E-mail addresses: zhxin@mail.buct.edu.cn (X. Zhang), hjxu@mail.buct.edu.cn (H. Xu)

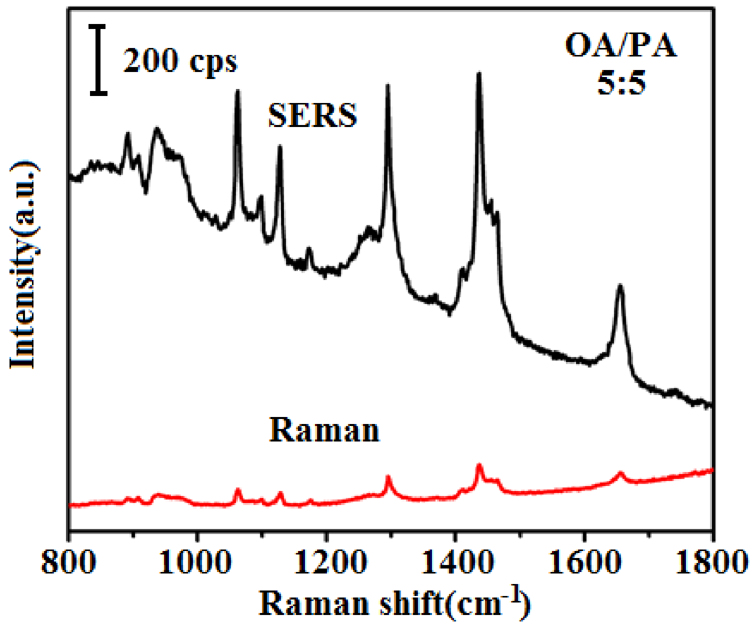

Figure S1. (a) SERS and (b) Raman spectra of OA/PA (5:5), tested on the Ag/Si substrate and Si wafer, respectively.

Verification of pure USFA S_{1656} values. The S_{1656} values of pure USFA, whose content is 100%, is added in Fig. 2(a), giving a new figure as shown in the following Fig. S2. It can be seen that the newly added data points conform to the extension cords of the original fitted detection working curves well. The S_{1656} values increase with the rise of the USFA content, and no saturation is observed even when the USFA content is 100%.

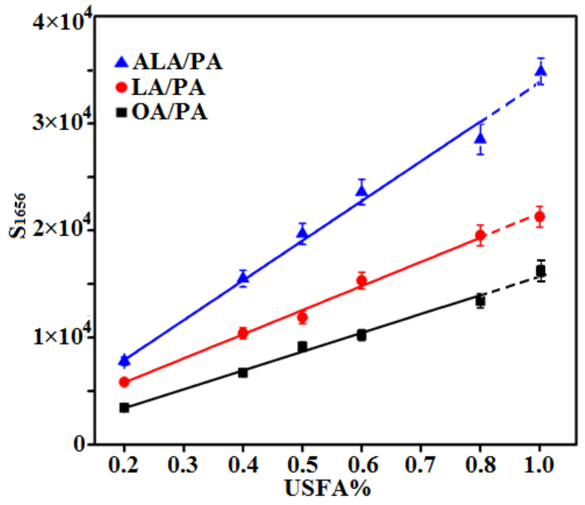

Figure S2. Detection working curve from 20% to 80% content, together with the added

S_{1656} values of pure USFA; the dotted lines are the extension cords of the working curves.

Characterizations of Ag/Si substrate. Figure S3a is a top FE-SEM image of the substrate, which shows clearly that a significant amount of Ag nanoparticles (NPs) with an average diameter of 400 nm were deposited on the Si nanowire (NW) array surface. The amplified image (Fig. S3b) shows that Ag NPs mainly adhered to the upper end of each Si NW. Figure S3c is a cross-section image of Ag/Si substrate, which reveals that the length and diameter of Si NWs are 13 um and 450 nm, respectively. Figure S3d is a TEM image of the substrate, which shows clearly that Ag NPs on the side of Si NW is smaller and sparse.

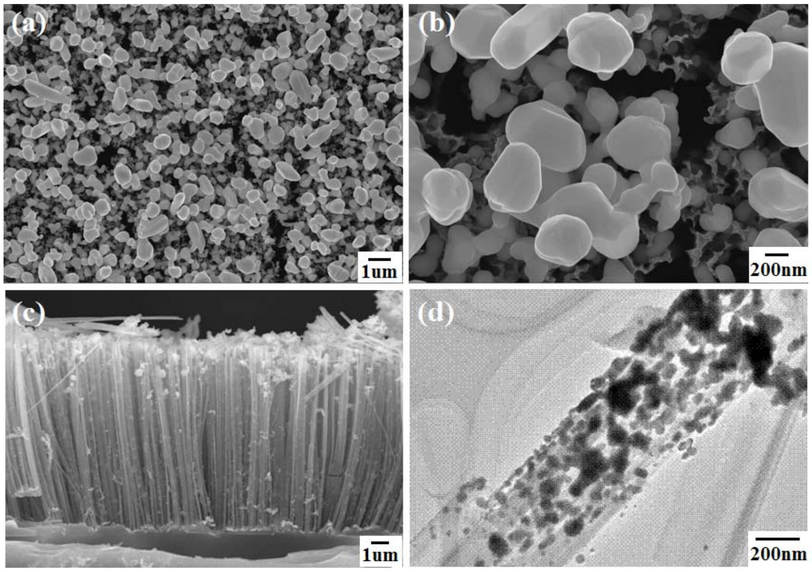

Figure S3. (a) Top, (b) magnified, (c) cross-sectional FE-SEM and (d) TEM images of Ag/Si substrate.

Table S1. Direct and normalized results of the S_{1656} values and the ETC of OA for the OA/ALA samples with different molar ratios

OA:ALA	a	a'	b	b'
2:8	12843	1.00	1.96	1.00
4:6	10812	0.84	1.72	0.88
5:5	9945	0.77	1.60	0.82
6:4	9482	0.74	1.48	0.76
8:2	8082	0.63	1.24	0.63

a: $S_{1656;}$ a': normalized values of $S_{1656;}$ b: ETC of OA; b': normalized ETC of OA.
